# Predictive accuracy of semi-quantitative scoring to screen for unfavorable ejection fraction and infarct size

**DOI:** 10.1186/1532-429X-13-S1-P44

**Published:** 2011-02-02

**Authors:** Lara Bakhos, Maria M Izquierdo-Gomez, Daniel C Lee, Edwin Wu

**Affiliations:** 1 Northwestern University, Chicago, IL, USA

## Background

Manual planimetry for quantitative analysis of ejection fraction (EF) and infarct size (IS) on cardiac magnetic resonance (CMR) imaging are too time-consuming, and therefore, impractical in daily clinical practice. We sought to derive and examine the predictive accuracy of a semi-quantitative scoring technique to screen patients with an EF ≤35% or an IS ≥18.5%, known independent predictors of increased cardiac events and mortality.

## Methods

The CMR derivation cohort consisted of 122 ST-segment elevation myocardial infarction patients. The validation cohort consisted of an additional 172 patients from the multi-center DEfibrillators To REduce Risk by MagnetIc ResoNance Imaging Evaluation (DETERMINE) trial. Cines were scored on a 17-segment model for wall motion and totaled for the Sum Motion Score (SMS): 0 = normal, 1 = mild hypokinesis, 2 = moderate to severe hypokinesis, 3 = akinesis, 4 = dyskinesis. Viability images were scored for infarct transmurality and totaled for the Sum Infarct Score (SIS): 0 = none, 1 = 1-25%, 2 = 26-50%, 3 = 51-75%, 4 = 76-100%. Quantitative EF and IS were manually planimetered using QMass (Medis).

## Results

From the derivation cohort, the SMS correlated with EF (R=-0.91, p<0.001) and SIS correlated with IS (R=0.94, p<0.001). Linear regression equations between SMS vs. EF and SIS vs. IS were obtained to estimate EF (est-EF = 55 - SMS) and IS (est-IS = 1.383 * SIS).

In the validation cohort, the mean EF was 39.0 ± 11.7% (32% with EF ≤35%), and the mean IS was 17.3 ± 10.4% (40% with IS ≤18.5%). Using the derivation formula, the est-EF (36.2 ± 10.9%) correlated with EF (R = 0.9) with a slight underestimation of the mean difference by 2.8 ± 4.9%. In addition, the est-IS (21.8 ± 12.8%) correlated with IS (R = 0.9). The est-IS tended to overestimate IS by 4.5 ± 5.9%. The sensitivity for detecting an EF ≤35% using the est-EF was 94% with a negative predictive value of 97%. The sensitivity for detecting an IS ≥18.5% using the est-IS was 96% with a negative predictive value of 96%.

## Conclusions

Semi-quantitative scoring is a sensitive screening tool that can be used to identify patients with an ejection fraction ≤35% or infarct size ≥18.5% to provide a rapid alternative method to manual planimetry.

